# A novel and well tolerated mite allergoid subcutaneous immunotherapy: evidence of clinical and immunologic efficacy

**DOI:** 10.1002/iid3.23

**Published:** 2014-05-29

**Authors:** Albert Roger, Nathalie Depreux, Yani Jurgens, Matthew D Heath, Gloria Garcia, Murray A Skinner

**Affiliations:** 1 Hospital Universitari Germans Trias Pujol, Unitat d'Al.lèrgiaBarcelona, Catalonia, Spain; 2 Allergy TherapeuticsEast Worthing, BN14 8SA, UK

**Keywords:** Allergen, allergic rhinitis, allergoid, cluster, conventional, cytokines, *Dermatophagoides pteronyssinus*, immunotherapy, nasal challenge test, specific immunoglobulins, subcutaneous

## Abstract

Allergy to house dust mite is one of the most common causes of allergic rhinitis. A novel tyrosine-adsorbed, modified allergen product, Acarovac Plus, developed for the treatment of perennial mite allergy seeks to address the underlying cause of allergic rhinitis in this instance. One of two dosing regimens may be used, either the Conventional Regimen or the Cluster Regimen. We sought to compare the efficacy and safety of a specific immunotherapy, developed for the treatment of perennial mite allergy, administered under a Conventional and Clustered dosing schedule in patients with persistent allergic rhinitis. Thirty adult patients, between 18 and 65 years old, with allergic rhinitis and/or asthma secondary to hypersensitivity to *Dermatophagoides pteronyssinus* were administered with either conventional or cluster initial regime, with a final visit one week after the last dose administration. The efficacy to the Conventional and Cluster regimens was measured using a Nasal Challenge Test monitoring clinical symptoms and peak nasal inspiratory flow. Total IgE, serum-specific inmunoglobulins (IgE and IgG4) to *Dermatophagoides pteronyssinus* and relevant cytokines (IFN-γ, IL-4, IL-5, IL-10 and IL-13) were assessed. A Satisfaction Questionnaire (TSQM) was completed after each patient's final visit. The tolerability of the vaccine was assessed monitoring adverse reactions. No adverse events were recorded in either conventional or cluster regime. The specific Nasal Challenge Test led to a decrease in symptom scores and a significant decrease in mean nasal peak inspiratory flow drop was recorded in both dosing regimen groups. A significant increase in IgG4-specific antibody titres was assessed. No significant changes were observed in concentrations of total IgE, specific IgE or cytokines (IFN-γ, IL-4, IL-5, IL-10 and IL-13). Patients declared themselves most satisfied in relation to “Secondary effects”, with high overall satisfaction in both groups. Cluster and conventional specific immunotherapy resulted in no adverse reaction(s) and led to similar improvements in immunological parameters, clinical efficacy (Nasal Challenge Test) and high overall satisfaction.

## Introduction

In Spain, allergic rhinitis is the symptom causing one in two of all patients to attend an allergy clinic for the first time. The prevalence in the general population is estimated to be between 10% and 25% and allergy to pollen and house dust mites are the most frequent diagnosis [1]. The prevalence of allergic disease is increasing and in Europe it is estimated that in 2015 that half the population will suffer from an allergic disease at some point in their lives [2]. One type of treatment is allergen-specific immunotherapy (SIT) which consists of administering (mostly by subcutaneous (s.c.) injections) the allergen(s) to an allergic person with the aim of increasing their immunological tolerance and decreasing the symptoms associated with subsequent exposure to the causative allergen(s) [3,4]. It is currently the only method of treatment that, as well as effectively decreasing allergic symptoms and the need for symptomatic medicine, also has the potential to alter the natural course of the disease [3,4].

Immunotherapy acts on T cells by changing the Th2 response and mucosal peripheral against allergens in favour of a Th1 response profile. This can be accompanied by a predominant allergen-specific IgG profile, especially the IgG4 isotype [3]. Recent studies have also identified changes in the pattern of cytokine secretion after immunotherapy, such as an increase in IL-10 [5,6]. In concert there can be changes in other cytokine profiles such as IL-4, IL-5, IL-13 and IFN-γ, highlighted in a number of different studies, both in vitro and in vivo [7,8].

Acarovac Plus is a novel tyrosine-adsorbed, modified allergen product developed for the treatment of perennial mite allergy. It contains extracts of selectively purified allergens from *Dermatophagoides pteronyssinus* (DPT). The native allergens are modified through treatment with glutaraldehyde and combined with l-tyrosine. Here, the glutaraldehyde will react with primary amines, facilitating cross-linking of the mix of proteins (allergen extract). The result of this is the formulation of a high molecular weight protein complex known as an allergoid [9,10]. It has been documented in specific immunotherapy that modified allergens (allergoids) are associated with reduced allergenicity, while maintaining immunogenicity (antigenic stimulation) [11–13]. Thus, the allergoid may be delivered, safely, in lower doses over a shorter period of time [10,14]. The modified allergen extract is then co-precipitated with l-tyrosine, which acts as a depot adjuvant when injected, causing the slow release of the allergoid in vivo – reducing the incident of local IgE-mediated inflammation [15]. Since l-tyrosine is a naturally occurring amino acid, it is biodegradable and rapidly removed from the injection site within 48 h post administration [15].

Acarovac Plus is an opaque white suspension for subcutaneous injection. SCIT has been used to treat house dust mite allergies for many years with proven clinical efficacy and safety [16–18]. A conventional up-dosing schedule will involve the patient receiving weekly injections, with only one injection being administered per visit. This is then followed by a dose-maintenance phase. Accelerated up-dosing schedules (cluster regimens) can also be administered, consisting also in weekly injections but with two doses per visit. Accelerated SIT build-up schedules, such as the cluster regimen, offer the advantage of reduced hospital visits, saving the patient time and expense [19]. The tolerance and safety of cluster regimens is well documented, whereas few studies have taken time to compare the efficacy and safety of conventional and cluster regimens with specific allergen/allergoid extracts [19]. Existing Mite vaccines have been used for rush dosing in Spain (in adults and children) and provided retrospective clinical and toxicological support to shape the rational design, formulation and dose regimen of Acarovac Plus.

## Materials and Methods

Starting in December 2012 and finishing in April 2013 a clinical study was conducted in the Allergy Unit in the Germans Trias i Pujol University Hospital, Barcelona Spain. The protocol was approved by the local Ethic Committee. The study was a prospective, observational, post-authorisation study of the safety, tolerability, and short-term effectiveness of Acarovac Plus with extract of DPT in adult patients with allergic rhinitis and/or asthma. The efficacy of the Conventional and Cluster regimens in the Acarovac Plus data sheet were measured using the Nasal Challenge Test, accompanied with a number of in vitro immunological tests measuring antibody titres and cytokine profiles involved in allergic reactions and immune responses to specific immunotherapy.

### Study materials

A therapeutic formulation comprised of *D. pteronyssinus* allergoid adsorbed to l-tyrosine has been designed, called Acarovac Plus. Following purification by clarification, the extract is diafiltered, modified with glutaraldehyde, diafiltered, sterile filtered, and then filled and analysed. All formulations were manufactured by Allergy Therapeutics (West Sussex, United Kingdom). A single dose is manufactured in one bottle which can cover one year's treatment for a patient. In the top dose vaccine, the calculated content of major allergen content of Der p 1 and Der p 2 is 4.8 and 0.16 µg/mL, respectively. The single strength of the product was rationally designed based on available in-house safety and efficacy studies. The Acarovac Plus is an opaque white suspension for injection that is administered subcutaneously in the upper arm.

### Trial design

The study population was made up of adult patients, between 18 and 65 years old, with allergic rhinitis and/or asthma secondary to hypersensitivity to DPT. Patients who were sensitised to other clinically relevant perennial or seasonal allergens, except other mites, patients who had received prior immunotherapy in the previous 5 years for the allergen tested, or patients with severe asthma were excluded. The duration of the study treatment was different according to the initial regime used, with a final visit one week after the last dose administration. Immunotherapy was administered either with a conventional or cluster regimens.

The Conventional Regimen is as follows: the Initial Phase administers the vaccine in increasing doses, starting the treatment with 0.05 mL and increase injections with an interval of 1 week between injections in steps of 0.05, 0.1, 0.3 mL until reaching 0.5 mL. The total duration of this schedule is 3 weeks.

The Cluster regimen cuts short the initial phase by administering more than one dose a day, as follows: the Initial Phase administers on Day 1 a first injection of 0.05 mL and 30 min later a second injection of 0.1 mL. Then a week later, on Day 8 a first injection of 0.1 mL and 30 min later a second injection of 0.3 mL. Then 3 weeks later, on Day 29 one injection of 0.5 mL. The total duration of this schedule is 4 weeks.

Statistically, categorical variables were described in terms of absolute and relative frequencies, including a confidence interval of 95%. To describe continuous variables, mean, standard deviation, median, minimum, and maximum were used, including the total number of valid values. Comparative tests of variables were used according to the nature of the variables. For quantitative variables, non-parametric tests were used (Mann–Whitney and Wilcoxon), according to the particular characteristics of the study variables. For qualitative variables a chi-squared test or a McNemar test were used. Statistical analyses were performed with the SAS statistical package, version 9.3.

### Nasal challenge test

The nasal challenge test consists of eliciting a response from the nasal mucosa by controlled exposure to the allergen, to evaluate the sensitivity to the allergen as well as the efficacy profile of drugs used to treat rhinitis [20]. The Nasal Challenge Test was carried out in the baseline visit and the final visit. The methodology followed was:

A baseline measure of peak inspiratory nasal flow was obtained, using three consecutive measurements and three values were recorded but the highest measure was considered for subsequent calculations. Then, each nostril was sprayed with a negative control. The patient was observed for 15 min during which time the number of sneezes was counted and the patient was asked for a score from 0 to 3 (0 = no symptoms, 1 = mild, 2 = moderate, 3 = severe) for his/her secretions, itching and nasal blockage. These values were recorded and the peak nasal inspiratory flow was measured again taking three consecutive measurements. Each nostril was sprayed again with the lowest concentration of the DPT allergen extract (DBU/mL) and the patient was observed for 15 min, during which time the number of sneezes was counted again, the patient was asked for a score for his/her secretion and nasal itching and the values were recorded. After 15 min three peak nasal inspiratory flow measures were taken again. This process was repeated by increasing the concentration of allergen extract each time, until a positive response was received.

A positive response was considered as:

- 5 or more sneezes or
- A drop in the peak nasal inspiratory flow greater than 50% (the highest value is always taken to calculate the % of the drop in peak nasal inspiratory flow).

### In vitro immunological measurements

The determination of total IgE and specific IgE to DPT was performed before starting immunotherapy and the Final Visit to assess changes in the degree of sensitization to the causative allergen of allergic disease. Specific IgG4 were analysed by enzyme immunoassay V0 and VF to test the immunogenicity of the extract and monitor the synthesis of specific blocking antibodies in response to immunotherapy. Analysis of cytokines involved in allergic reactions and immune response to specific immunotherapy (IFN-γ, IL-4, IL- 5, IL-10, IL-13) was conducted to assess the induction of Th1 and T-regulatory. Detection of total IgE and specific IgE (normal < 0.35 kU/L) and IgG4 specific (mgA/L) in serum was performed using ELiSA™ (ImmunoCAP®; Phadia AB, Uppsal, Sweden) using the 250® analyzer ImmunoCAP (Phadia AB).

Cytokine levels in serum were measured by Cytometric bead array (CBA) (Becton Dickinson) Biosciences, San Jose, CA, USA), the levels of IFN-γ, IL-4, IL-5, IL-10 and IL-13 were measured. Samples were acquired on a flow cytometer (LSR Fortessa, BD Biosciences).

### TSQM questionnaire

The Treatment Satisfaction Questionnaire for Medication has been validated in a study by Atkinson et al. [21]. The conclusion was reached that the TSQM is a psychometrically sound and valid measure of the major dimensions of patients' satisfaction with medication. Preliminary evidence suggests that the TSQM may also be a good predictor of patients' medication adherence across different types of medication and patient populations. The results of a recent study of the Spanish version of the TSQM by Trujols et al., strongly suggest the TSQM value as a brief, generic, and psychometrically sound instrument to assess satisfaction with a medication in a multidimensional manner [22]. In the present study TSQM was performed during Final Visit.

## Results

A total of 30 patients were recruited, of whom 15 followed the conventional regimen and the remainder the cluster regimen. 40% of the patients included were men and the average age of the population was 35.2 years (SD = 10.7). None of the patients on the conventional regimen or the cluster regimen suffered adverse reactions.

## Nasal Challenge Test: Response Scores

Patients who received conventional and cluster dosing regimen were assigned to baseline groups who were exposed to different concentrations of DPT allergen extracts (50, 500, 5000 or 50,000 DBU/mL, respectively). The Nasal Challenge Test was carried out at each baseline visit and the result(s) of the tests compared with the final visit, in which each patient received a final dose (50–50,000 DBU/mL), outlined in Supplementary Table S1. A positive response was considered as:

- 5 or more sneezes or
- A drop in the peak nasal inspiratory flow greater than 50% (the highest value is always taken to calculate the % of the drop in peak nasal inspiratory flow).

A decrease in positive symptoms was observed in all patients exposed between 50 and 50,000 DBU/mL DPT allergen extract. At the lowest dose concentration (50 DBU/mL), a statistically significant decrease (McNemar test; *P* < 0.05) was observed in the proportion of patients in whom the nasal challenge test was positive (23.3% at the baseline visit and 3.3% at the final visit) (Supplementary Table S1). Only one patient (3.3%) had a negative result at the final visit.

## Nasal Challenge Test: Symptom Scores

**Figure 1:**
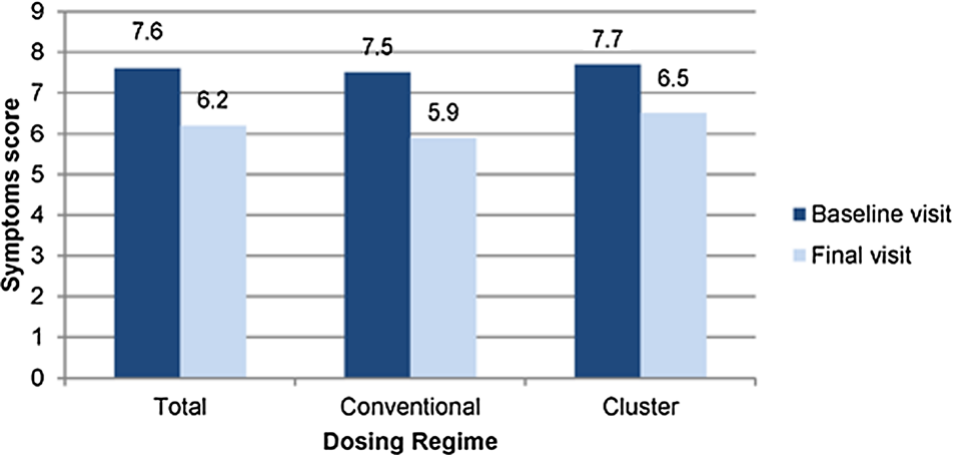
Patient symptoms scores at baseline and final visits. Symptom scores (0–9) are based on the sum of the three symptoms (sneezing, secretion and nasal itching).

During the nasal challenge test, symptom scores of each patient were recorded at the baseline and final visits, respectively. The scores shown are those recorded from patients, which exhibited a positive result at the baseline visit. During which time the number of sneezes was counted and the patient was asked for a score for his/her secretion and nasal itching. A significant improvement in symptom scores were recorded from patients who received therapy using either a conventional or cluster dosing regimen (Fig. 1). No statically significant difference was observed between both cluster and conventional regimes.

## Nasal Challenge Test: Peak Nasal Inspiratory Flow

**Figure 2:**
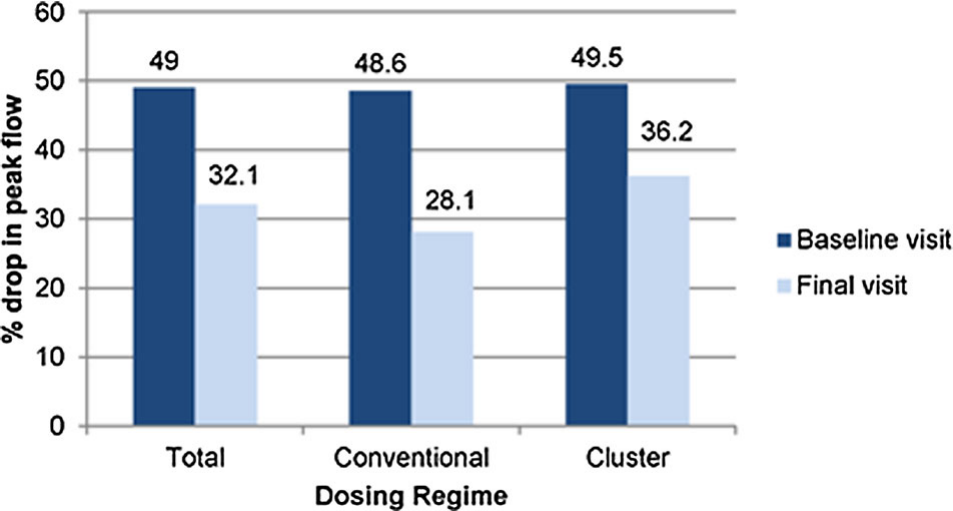
Mean PIFR of patients while carrying out the nasal challenge test, at baseline and final visit(s).

The mean peak nasal inspiratory flow rate (PIFR) of patients was recorded while carrying out the nasal challenge test at the baseline and final visit, respectively (Fig. 2). At the baseline visit, the mean decrease in peak flow was 49.0% (SD = 10.4) and at the final visit it was 32.1% (SD = 18.4), exhibiting a statistically significant decrease (−16.89%) (Wilcoxon test; *P* < 0.05). No statically significant difference was observed between both cluster and conventional regimes.

## Immunology

The concentration(s) of specific IgG4 to DPT, total IgE, specific IgE, and cytokines involved in allergic reactions and immune responses to specific immunotherapy (IFN-γ, IL-4, IL-5, IL-10 and IL-13) were measured before starting immunotherapy (baseline) and after their final visit (Table1).

**Table 1:** Evolution of IgG4

Specific IgG4	Visit	*n*	Mean	SD	Median	Min.	Max.	*P*-value^1^
Total	Baseline visit	30	0.5	0.5	0.3	0.1	2.1	–
	Final visit	29	0.6	0.6	0.5	0.1	2.5	<0.0001
Conventional	Baseline visit	15	0.6	0.6	0.3	0.1	2.1	–
	Final visit	15	0.7	0.7	0.3	0.1	2.5	0.0060
Cluster	Baseline visit	15	0.4	0.2	0.4	0.1	0.7	–
	Final visit	14	0.6	0.3	0.5	0.2	1.0	0.0002

1 Wilcoxon test; *P* < 0.05.

The evolution of study patients' specific IgG4 immunology exhibited a statistically significant increase at the final visit, with respect to the baseline visit (Wilcoxon test; *P* < 0.05), both in the global sample and among patients on the conventional or cluster regime (Table1).

No significant changes were observed in concentrations of total IgE, specific IgE or cytokines (IFN-γ, IL-4, IL - 5, IL-10 and IL-13).

## TSQM Survey

**Figure 3:**
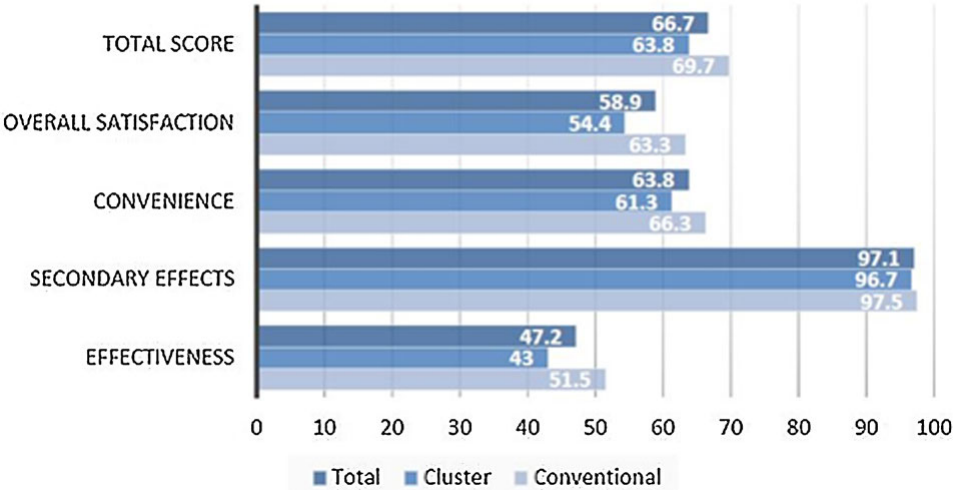
Categorised TSQM question scores (Satisfaction range 0–100; 0 = not at all, 100 = very strong).

A TSQM was conducted with patients after their final visits (Fig. 3). The analysis of the questionnaire revealed “Secondary effects” and “Convenience,” with mean scores of 97.1 (SD = 7.8) and 63.8 (SD = 12.9), respectively, as the highest mean scores recorded. This was followed by “effectiveness” (47.2, SD = 19.2) and “overall satisfaction” (58.9, SD = 19.4). Total mean score for the TQSM questionnaire was 66.7 (SD = 11.55). There was no significant differences in scores between conventional and cluster therapy groups (Mann–Whitney *U*-test; *P* > 0.05).

## Discussion

In this study, patients received immunotherapy using a cluster or conventional dosing regimen of Acarovac Plus; a novel tyrosine-adsorbed, modified allergen product (with extract of DPT) developed for the treatment of perennial mite allergy. It has been documented in specific immunotherapy that modified allergens (allergoids) are associated with reduced allergenicity, while maintaining immunogenicity (antigenic stimulation) [11–13]. Thus, the allergoid may be delivered, safely, in lower doses over a shorter period of time [10,14]. The cluster schedule is known to be a safe and effective alternative to the conventional schedule in both children and adults. In addition to this, it has the advantage of considerably reducing the duration of the initiation phase of subcutaneous and sublingual immunotherapy. Nevertheless, this form of administering the treatment must be carried out in specialist allergy units and not in primary care, where the patient would be sent, subsequently, for the corresponding maintenance doses. A comparative study of cluster (6-weeks) and conventional (14 weeks) immunotherapy schedules with *D. pteronyssinus*, in the treatment of allergic rhinitis, was performed previously [19]. Here, similar improvements in clinical symptoms and quality of life from patients were observed in both schedules. Thus, provides clear evidence that the cluster schedule is not only a safe and efficacious alternative, but also allowing its clinical effectiveness to arrive sooner for the patient [19]. In this study, we used the cluster immunotherapy up-dosing schedule, entailing five injections in three visits, compared with the conventional schedule, which administered four injections in four visits. The Nasal Challenge Test was conducted with patients from each dosing regimen and was designed to assess the evolution of clinical symptoms, improvements in quality of life (symptom scores) and immunological parameters.

The assessment of clinical symptoms was first compared by measuring responses (from baseline and final visits) of the nasal mucosa after controlled exposure to the DPT allergens. Both cluster and conventional specific immunotherapy led to a similar decrease in positive response scores in all patients exposed between 50 and 50,000 DBU/mL DPT allergen extract at the baseline and final visits. This was significant in patients exposed to 50 DBU/mL. Similar improvements in symptom scores were observed in both groups. This was further supported by the quantitative decrease in PIFR in subjects at baseline and final visits (Fig. 2), highlighting the clinical effectiveness of treatment of patients in this cohort. It is well documented that nasal congestion is correlated with restricted lower nasal airflow function in adults with seasonal rhinitis [23,24]. Key studies have demonstrated that PIFR measurements reflect the severity of nasal signs of rhinitis in a general population sample of young adults [24]. The strong association between PFIR and rhinitis signs in this study provides objective and complementary evidence of the decrease in response and symptom scores of the patient, during nasal challenge testing. This reflects good specificity and a high negative predictive value for moderate/sever signs of rhinitis.

Serum concentration of allergen-specific IgG has been shown to correlate with clinical improvements. The induction of IgG antibodies, induced by sub-cutaneous immunotherapy (SCIT), has been shown to reduce allergic inflammation. This can be achieved through the reduction of IgE-mediated degranulation of mast cells or basophils [25,26] via a number of different pathways [25,26]. One such pathway can involve the “blocking” of allergen-specific IgE, as a result of competitive binding with IgG against the same epitopes. Another pathway can involve initiation of inhibitory signals triggered through low affinity IgG receptors (FcγRIIB) on mast cells and basophils. For example, it has been demonstrated that blood from subjects who received immunotherapy contain IgG antibodies that mediate inhibition of basophil activation by a mechanism that is blocked by antibodies specific for the inhibitory IgG receptor FcγRIIB [26]. Zhang et al., together with other studies, also describe no change in specific IgE after 1 year SCIT treatment in either cluster or conventional schedules. In this study, significant increases in IgG4 antibody-specific titre (Table1), while maintaining levels of total and specific IgE and allergy-linked cytokine profiles were recorded at baseline and final visits. This highlights the potential functional activity induced following SCIT in this instance. The presence of such cytokine profiles are widely documented during allergen immunotherapy [26]. For example, IL-10 may mediate the decrease in antigen-specific IgE production and increase IgG4 levels, while dampening down a production in pro-inflammatory substances from mast cells, eosinophil's and T cells. IFN-γ is known to inhibit Th2 cell differentiation [26]. The introduction of SCIT treatment is thought to prevent any increase in pro-inflammatory cytokines (IL-4, -5 and -13), which was recorded herein.

The absence of any adverse reactions is reflected in the TSQM survey where patients declared themselves most satisfied in relation to “Secondary effects” (97.1) (Fig. 3). Nasal congestion is widely documented as the most severe and predominant symptom which patients most want to prevent from occurring, severely impacting their quality of life [23]. The range of pharmacologic options (e.g., antihistamines, oral decongestants) for nasal congestion in allergic rhinitis is generally considered to be minimally effective in prevention or as long-term treatment [23]. The Nasal Challenge Test provides distinct consideration of these parameters through qualitative, quantitative and objective means. Overall, patients awarded a total satisfaction score of 66.7 and while no significant differences were observed between Cluster and Conventional dosing schedules, patients will undoubtedly achieve clinical effectiveness with fewer visits under a cluster dosing schedule—in addition to providing a safe alternative dosing regimen.

## Conclusion

Cluster and conventional specific immunotherapy led to similar decreases in clinical symptoms and improvements in immunological parameters. None of the patients on the conventional regimen or the cluster regimen suffered adverse reactions and overall satisfaction from TSQM surveys was high.

## SUPPORTING INFORMATION

**Table S1:** Nasal challenge test: response scores.
