# Supplementary material for: A novel and well tolerated mite allergoid subcutaneous immunotherapy: evidence of clinical and immunologic efficacy
Source: Immun Inflamm Dis. 2014 May 29;2(2):92–8. doi: 10.1002/iid3.23 (PMC4217550; doi:10.1002/iid3.23)
Supplement: Supplementary file 1 — Table S1: Nasal challenge test: response scores. [file iid30002-0092-SD1.docx]

**Table S1:** Nasal challenge test: response scores

| **Allergen conc. (DBU/mL)** | **Conventional** | | | **Cluster** | | | **Total** |  |  |
| --- | --- | --- | --- | --- | --- | --- | --- | --- | --- |
|  | **n** | **%^1^** | **%^2^** | **n** | **%^1^** | **%^2^** | **n** | **%^1^** | **%^2^** |
| Baseline: 50 DBU/ml | 6 | 40.0 | **100.0** | 1 | 6.7 | **100.0** | 1 | 6.7 | **100.0** |
| Final: 50 DBU/ml | 1 | 6.7 | 16.7 | 0 | 0.0 | 0.0 | 0 | 0.0 | 0.0 |
| Final: 500 DBU/ml | 5 | 33.3 | 83.3 | 1 | 6.7 | 100.0 | 1 | 6.7 | 100.0 |
| Baseline: 500 DBU/ml | 6 | 40.0 | **100.0** | 5 | 33.3 | **100.0** | 5 | 33.3 | **100.0** |
| Final: 500 DBU/ml | 4 | 26.7 | 66.7 | 2 | 13.3 | 40.0 | 2 | 13.3 | 40.0 |
| Final: 5000 DBU/ml | 2 | 13.3 | 33.3 | 3 | 20.0 | 60.0 | 3 | 20.0 | 60.0 |
| Baseline: 5000 DBU/ml | 3 | 20.0 | **100.0** | 7 | 46.7 | **100.0** | 7 | 46.7 | **100.0** |
| Final: 500 DBU/ml | 0 | 0.0 | 0.0 | 1 | 6.7 | 14.3 | 1 | 6.7 | 14.3 |
| Final: 5000 DBU/ml | 2 | 13.3 | 66.7 | 6 | 40.0 | 85.7 | 6 | 40.0 | 85.7 |
| Final: 50000 DBU/ml | 1 | 6.7 | 33.3 | 0 | 0.0 | 0.0 | 0 | 0.0 | 0.0 |
| Baseline: 50000 DBU/ml | 0 | 0.0 | **0.0** | 2 | 13.3 | **100.0** | 2 | 13.3 | **100.0** |
| Final: 50000 DBU/ml | 0 | 0.0 | 0.0 | 1 | 6.7 | 50.0 | 1 | 6.7 | 50.0 |
| Final: test negative | 0 | 0.0 | 0.0 | 1 | 6.7 | 50.0 | 1 | 6.7 | 50.0 |

^1^ Percentage (positive responses) calculated on total assessable patients.

^2^ Percentage (positive responses) calculated on each of the baseline concentrations.
